# Potential pathways by which maternal second-hand smoke exposure during pregnancy causes full-term low birth weight

**DOI:** 10.1038/srep24987

**Published:** 2016-04-29

**Authors:** Zhongzheng Niu, Chuanbo Xie, Xiaozhong Wen, Fuying Tian, Shixin Yuan, Deqin Jia, Wei-Qing Chen

**Affiliations:** 1 Department of Biostatistics and Epidemiology, School of Public Health, Sun Yat-sen University, Guangzhou, China; 2 School of Community and Global Health, Claremont Graduate University, Claremont, CA, USA; 3 Division of Behavioral Medicine, Department of Pediatrics, School of Medicine and Biomedical Sciences, State University of New York at Buffalo, Buffalo, USA; 4 Shenzhen Women and Children’s Hospital, Shenzhen, China; 5 Foshan Women and Children’s Hospital, Foshan, China

## Abstract

It is well documented that maternal exposure to second-hand smoke (SHS) during pregnancy causes low birth weight (LBW), but its mechanism remains unknown. This study explored the potential pathways. We enrolled 195 pregnant women who delivered full-term LBW newborns, and 195 who delivered full-term normal birth weight newborns as the controls. After controlling for maternal age, education level, family income, pre-pregnant body mass index, newborn gender and gestational age, logistic regression analysis revealed that LBW was significantly and positively associated with maternal exposure to SHS during pregnancy, lower placental weight, TNF-α and IL-1β, and that SHS exposure was significantly associated with lower placental weight, TNF-α and IL-1β. Structural equation modelling identified two plausible pathways by which maternal exposure to SHS during pregnancy might cause LBW. First, SHS exposure induced the elevation of TNF-α, which might directly increase the risk of LBW by transmission across the placenta. Second, SHS exposure first increased maternal secretion of IL-1β and TNF-α, which then triggered the secretion of VCAM-1; both TNF-α and VCAM-1 were significantly associated with lower placental weight, thus increasing the risk of LBW. In conclusion, maternal exposure to SHS during pregnancy may lead to LBW through the potential pathways of maternal inflammation and lower placental weight.

Low birth weight (LBW, birth weight <2500 g) may result from preterm birth or intrauterine growth restriction (IUGR), which can occur simultaneously in pregnancy^1^. There is accumulating evidence that LBW is not only the strongest single risk factor for perinatal, neonatal and infant mortality^2^, but is also related to neurological and behavioural problems in childhood^3^,^4^ and chronic diseases in adulthood^5^,^6^. Therefore, researchers have started paying attention to studies on the etiological factors associated with LBW and the potential pathophysiological mechanisms.

It is well documented that maternal exposure to second-hand smoke (SHS) is an important risk factor for LBW. A recent meta-analysis estimated that maternal exposure to SHS during pregnancy reduces mean birth weight by 31–60 grams and may increase the risk of LBW by 1.16–1.60 times^7^. However, the biological mechanisms by which maternal exposure to SHS during pregnancy causes LBW have not been established. A potential pathway may be that maternal exposure to SHS during pregnancy causes maternal inflammation, which disrupts the placenta’s ability to transfer sufficient nutrients and oxygen to the foetus, and finally results in LBW.

The aforementioned hypothesis is suggested by the following strands of evidence. First, SHS exposure leads to abnormal levels of inflammatory markers in different populations^8^,^9^. Second, the elevated maternal inflammatory markers are independently related to LBW^10^ and mediate the associations between periodontal disease^11^, maternal sleep disturbances during pregnancy^12^, maternal pre-pregnancy body mass index (BMI)^13^ and LBW. Third, the placenta is associated with birth weight and IUGR^14^,^15^,^16^. Fourth, maternal smoking during pregnancy impairs the structure and functioning of the placenta^17^. Fifth, placental weight partially mediates the effects of prenatal factors such as pre-pregnancy obesity, gestational diabetes mellitus and excessive gestational weight gain on foetal growth^18^. Sixth, abnormal inflammatory markers have been found in the placentas of LBW cases^19^.

To the best of our knowledge, there is no research that links the above evidence together to explore the plausible mechanisms. As such, this study aimed to explore the potential pathways that integrate maternal inflammation and the placenta, which might explain the mechanism by which maternal exposure to SHS during pregnancy leads to LBW.

## Results

### Social-demographic and obstetric characteristics of participants

Table 1 compares the socio-demographic and obstetric characteristics of the full-term LBW and control participants. Apart from their significantly lower birth weight (2343.87 g vs 3300.99 g), full-term LBW infants had a shorter gestational age (38.03 weeks vs 39.08 weeks) and were more likely to be female (59.8% vs 48.2%). Other characteristics, including maternal age, ethnicity, marital status, education level, pre-pregnancy BMI and parity were quite comparable between the full-term LBW cases and the controls.

### Associations between maternal exposure to SHS, lower placental weight, maternal serum inflammatory markers and full-term LBW

Table 2 presents the results of the associations between maternal exposure to SHS exposure, lower placental weight, maternal serum inflammatory markers and full-term LBW. After controlling for potential confounders, maternal SHS exposure was significantly and positively associated with lower placental weight (adjusted OR = 2.30; 95% CI = 1.10–4.81), increased maternal TNF-α (adjusted OR = 1.55; 95% CI = 1.06–2.27) and IL-1β (adjusted OR = 1.72; 95% CI = 1.11~2.66). There were also significant and positive associations between lower placental weight and increased maternal TNF-α (adjusted OR = 1.28; 95% CI = 1.01–1.63) and IL-1β (adjusted OR = 1.35, 95% CI = 1.00–1.80).

After controlling for potential confounders, significant positive correlations were observed between MCP-1 and IL-6 (partial relationship, *pr* = 0.19), IL-6 and CRP (*pr* = 0.11), TNF-α and IL-1β (*pr* = 0.25), TNF-α and CRP (*pr* = 0.12), TNF-α and VCAM-1 (*pr* = 0.14), IL-1β and CRP (*pr* = 0.11) and CRP and VCAM-1 (*pr* = 0.12), whereas significant negative correlations were obtained between IL-6 and TNF-α (*pr* = −0.14), IL-6 and VCAM-1 (*pr* = −0.14).

Furthermore, after controlling for potential confounders, full-term LBW was significantly and positively associated with maternal SHS exposure during pregnancy (adjusted OR = 2.14; 95% CI = 1.06–4.32), lower placental weight (adjusted OR = 3.60; 95% CI = 2.09–6.19) and increased maternal serum levels of TNF-α (adjusted OR = 1.92; 95% CI = 1.47–2.50) and IL-1β (adjusted OR = 1.53; 95% CI = 1.14–2.05)

### Potential pathways by which maternal SHS exposure during pregnancy causes full-term LBW

**Figure 1:**
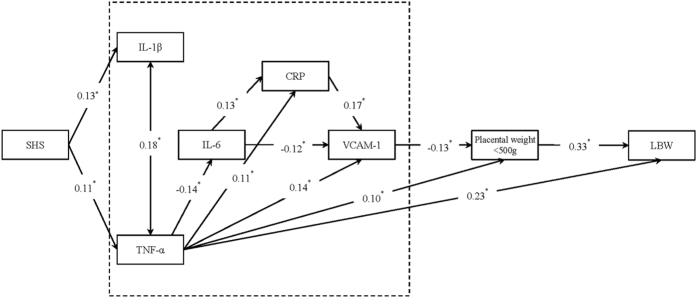
The potential pathways of maternal secondhand smoke exposure during pregnancy, maternal serum inflammatory markers, placental weight, and low birthweight at term. Note: A one-way arrow linked from influential factor to the response factor. Double headed arrow indicated a correlation between TNF-α and IL-1β Parameters on the path represented direct effects. Only significant effects were kept in this figure and were marked with an asterisk.

The SEM results indicated a good fit to the model (GFI = 0.981, AGFI = 0.947, CFI = 0.988, IFI = 0.989, RMSEA = 0.029). The coefficients in the pathway diagrams indicated that maternal SHS exposure during pregnancy might induce full-term LBW indirectly (Fig. 1). First, maternal SHS exposure during pregnancy led to maternal inflammation by increasing the secretion of TNF-α and IL-1β, which further triggered the secretion of IL-6, CRP and VCAM-1. Second, TNF-α and VCAM-1 in turn affected placental weight. Third, TNF-α and lower placental weight directly affected the full-term LBW.

The effect of maternal SHS exposure on full-term LBW via each pathway was calculated, as shown in Table 3. There were two indirect pathways, one from IL-β to the placenta (path 2, effect = 0.031, proportion to total effect = 24.2%), and the second from IL-1β to TNF-α (path 3, effect = 0.031, proportion to total effect = 24.2%). Thus, the combined mediation effect of the two pathways was 0.128, and the proportion of the total effect was 48.4% (24.2% + 24.2%).

## Discussion

This study tested the hypothesis that maternal SHS exposure during pregnancy leads to full-term LBW through maternal inflammation and lower placental weight. The SEM results showed that maternal exposure to SHS during pregnancy initially induced maternal inflammation by increasing the secretion of IL-1β and TNF-α, which then triggered the secretion of IL-6, CRP and VCAM-1; both TNF-α and VCAM-1 caused a decrease in placental weight; and eventually, TNF-α and the damaged placenta increased the risk of full-term LBW.

Previous studies have reported inconsistent associations between SHS exposure and the six measured inflammatory markers in our study. For example, two studies of male workers reported that SHS exposure was significantly associated with elevated serum CRP^9^,^20^, while two further studies in adults^8^,^21^ and one in adolescents^22^ found a non-significant relationship between SHS exposure and serum CRP. Our study showed an insignificant association between maternal SHS exposure during pregnancy and maternal serum CRP levels. Regarding the associations between SHS exposure and three pro-inflammatory markers, IL-1β, IL-6 and TNF-α, Wilson *et al.* found that healthy children exposed to SHS had lower serum concentrations of IL-1β than those without SHS exposure^23^, but an inverse association was observed in adults^24^ and an insignificant association in adolescents was reported by Matsunaga *et al.*^22^. Five studies found that SHS exposure had no significant effect on serum IL-6 in adolescents^22^,^23^ and adults^9^,^21^,^24^, while a study in the elderly reported a significant association between SHS and elevated serum IL-6 levels^25^. An insignificant association between SHS exposure and serum TNF-α was reported by four studies, two in adolescents^22^,^23^ and two in adults^9^,^24^, and a positive association was reported by another study in adults^24^. Our study found that maternal exposure to SHS during pregnancy was significantly associated with elevated serum levels of TNF-α and IL-1β but not IL-6. There is little information on the relationship between SHS exposure and serum VCAM-1. Only one study, by Matsunaga *et al.*, has reported an insignificant association between them in adolescents^22^, and a similar result was observed in our study. To the best of our knowledge, there has been no report on the effect of SHS exposure on serum MCP-1 levels. Nevertheless, two studies in aged persons^25^ and healthy adults^26^ reported that active smokers had higher serum MCP-1 concentrations than never smokers, and Garliches *et al.* found that young healthy male smokers had slightly decreased serum MCP-1 levels^27^. However, there was no significant difference between the serum MCP-1 levels of pregnant women with and without SHS exposure in our study. Taken as a whole, the previous discrepant results on inflammatory markers may be explained by differences in the duration and extent of SHS exposure, and the age, gender, biological status and ethnicity of the subjects in different studies^22^. Therefore, further studies are needed to clarify the effects of SHS exposure on inflammatory markers in pregnant women.

There is abundant evidence showing that maternal active smoking during pregnancy profoundly alters placental weight, morphology and function^17^,^28^,^29^,^30^. However, there is little research on how maternal exposure to SHS during pregnancy affects the placenta. A study by Rath *et al.* indicated that maternal exposure to SHS in pregnancy could produce similar ultra-structural changes as maternal active smoking^30^, while another study by Ramesh *et al.* observed no significant differences in placental surface area, volume and weight between pregnant women with and without SHS exposure^31^. However, our study found that pregnant women exposed to SHS had a lower placental weight than those not exposed to SHS. These discrepancies may due to differences in the duration and extent of maternal exposure to SHS during pregnancy and the ethnicity of subjects.

Although the association between LBW and inflammation has been explored, the results varied for the different kinds of inflammatory markers and with the period of pregnancy in which the specimens were collected. With regards to the association between maternal serum TNF-α level and LBW, three studies reported that pregnant women with LBW had significantly higher serum TNF-α levels before delivery than those with normal birth weight^32^,^33^,^34^. Similarly, our study showed that a high maternal serum TNF-α level before delivery significantly increased the risk of full-term LBW. A study by Amu *et al.* found significantly elevated serum IL-1β levels in women who delivered with IUGR^35^. Kalinderis *et al.* reported the same result in women with LBW and complications with pre-eclampsia^36^. Elfayomy *et al.* found that women with full-term IUGR had significantly higher serum IL-6 levels than women with babies appropriately grown for gestational age (AGA)^31^, and Steenwinkel *et al.* reported that maternal serum IL-6 levels during the first trimester were significantly associated with poor foetal growth in pregnant women with rheumatoid arthritis^37^. In line with the findings of two other studies^32^,^34^, we discovered a non-significant association between maternal serum IL-6 and full-term LBW. To date, there are no consistent reports on the association between maternal serum CRP level and LBW. For instance, Tjoa *et al.* reported that an elevated maternal serum CRP level during the first trimester of pregnancy significantly increased the risk of delivering a growth-restricted baby^38^. In contrast, maternal serum CRP level in the second trimester was not significantly associated with IUGR^39^, and a non-significant difference in maternal serum CRP before delivery was observed between pregnant women with IUGR and AGA^40^. Similarly, our study showed no significant differences between the maternal serum CRP levels of women with LBW and normal birth weight. There are limited reports on the associations between LBW and maternal serum levels of MCP-1 and VCAM-1. To the best of our knowledge, only two studies have found maternal serum MCP-1 levels to be lower in women with IUGR than control groups^41^,^42^, and one study found an insignificant difference between the maternal serum VCAM-1 levels of SGA cases and controls^43^. However, our study did not find significant associations between full-term LBW and maternal serum levels of MCP-1 and VCAM-1. To the best of our knowledge, there are no published studies on the relationship between maternal serum IL-1β and LBW. Interestingly, we found a significant positive association between them. We assumed that the discrepancies in the results of these studies might be caused by differences in the period of pregnancy in which samples were taken and the ethnicity of subjects.

The association between placental weight and birth weight is well established. Previous studies provide consistent evidence that placental weight is much lower in LBW cases than in control groups with normal birth weight^14^,^15^,^16^. Another study found that other placental gross indicators, such as the minor axis of the placenta surface, were sensitive to the mother’s nutritional state and foetal nutritional demands^44^. In accordance with these findings, our study found that lower placental weight was significantly associated with the risk of full-term LBW.

In line with previous findings^7^, our study also found that SHS exposure during pregnancy significantly increased the risk of full-term LBW after controlling for potential confounding factors. Our study indicated two potential pathways by which maternal SHS exposure during pregnancy might cause full-term LBW. First, maternal exposure to SHS increased maternal systemic IL-1β and TNF-α, which then triggered the secretion of IL-6, CRP and VCAM-1. Subsequently, both TNF-α andVCAM-1 caused a decrease in placental weight, which had a direct effect on foetal weight. Second, maternal SHS exposure induced maternal secretion of TNF-α, which could cross the placenta and directly increase the risk of full-term LBW. Similarly, Ouyang *et al.* found that placental weight mediated the effects of prenatal factors on foetal growth^18^, and several other studies have indicated that increased maternal systematic inflammation mediated the association of periodontal disease^11^, maternal sleep disturbances during pregnancy^12^ and pre-pregnancy BMI^13^ with adverse pregnancy outcomes. Taken together, these findings imply that maternal inflammation and its effect on the placenta may serve as mediators in the process by which maternal prenatal exposure to SHS and other factors affect foetal growth and lead to full-term LBW.

As a critical maternal inflammatory marker in our study, TNF-α is a pleiotropic pro-inflammatory cytokine, which may have direct effects on the placenta. Previous studies have suggested that an excessive level of TNF-α may damage the placenta by inhibiting the growth of the trophoblast, causing apoptosis of trophoblast cells, interfering with the development and invasion of placental spiral arteries and damaging the endothelium and decidual vasculature^45^,^46^. Elevated maternal serum levels of TNF-α were found in IUGR cases with placental insufficiency but not in normal pregnancy^46^,^47^, which could explain our finding that TNF-α indirectly affected full-term LBW by decreasing placental weight. In addition, a study found that TNF-α can transfer through the placenta and infiltrate the foetal circulation^48^. TNF-α may be involved in the metabolic regulation of glucose, lipids and insulin resistance by down-regulating insulin-stimulated glucose uptake and affecting the glucose transporter, the insulin receptor autophosphorylation and the insulin receptor substrate-1, all of which tend to reduce lipid accumulation within adipose tissue^49^,^50^. This may explain the other potential pathway through which TNF-α can cause full-term LBW without any decrease in placental weight in this study.

Our results revealed a noteworthy role of VCAM-1. As a marker of endothelial cell activation, VCAM-1 is a crucial adhesion molecule in both efficient placentation and adequate placental vasculature development^51^. A previous study found that low levels of VCAM-1 could be partly responsible for defects in trophoblastic cells, poor endovascular invasion and a lack of epithelial-endothelial transformation^52^, all of which contribute to the pathological basis of LBW. Utero-placental deficiency has also been attributed to decreased VCAM-1 expression in cases of foetal growth restriction^53^. Moreover, TNF-α and IL-1β can stimulate human endothelial cells to express VCAM-1^54^,^55^. All of these findings may explain our finding that maternal VCAM-1 level was directly associated with maternal serum TNF-α, IL-6 and CRP levels, and indirectly with IL-1β via TNF-α. Taken together, these results indicate that VCAM-1 may play a crucial mediation role in the associations between maternal serum inflammatory cytokines and placental functioning, and indirectly affect full-term LBW by influencing placental weight.

We explain the biological mechanisms of maternal exposure to SHS during pregnancy resulting in full-term LBW as follows. First, some of the components (such as carbon monoxide, nicotine, cadmium, etc.) in SHS may cause a maternal inflammatory response. Then, some inflammatory cytokines (such as TNF-α, IL-1β and VCAM-1) may damage the placenta or affect placental development resulting in deficiencies in its size, structure and function; finally, the damaged or insufficiently developed placenta is unable to transfer sufficient nutrients and oxygen from the maternal body to the foetus, which may eventually lead to full-term LBW.

Several limitations should be addressed. First, all of the subjects were sampled from women and children’s hospitals in this study, which might have caused a selection bias that would limit the generalisability of our findings. Second, maternal SHS exposure was retrospectively assessed using a questionnaire that might have introduced information bias and confused the association between maternal SHS exposure during pregnancy and full-term LBW. Third, we only measured placental weight, while other physical parameters of the placenta, such as the placental width and length and the cord placement, may also predict outcomes. Fourth, inflammation is an active process involving the release of an extensive array of inflammatory markers, yet this study only included six markers. Therefore, further research should take a ‘systematic biology’ approach to explore how disturbances in inflammatory networks may lead to full-term LBW. Fifth, we only obtained maternal serum at one time point, near parturition, which limited our ability to define the temporal changes in these cytokine profiles and weakened the clinical application value when attempting to predict adverse pregnancy outcomes early in gestation. Parturition may also increase the level of inflammatory cytokines such as IL-1β, and thus the association between inflammatory markers and full-term LBW may be more complicated than our simplified explanation. Although we controlled for gestational age as a potential confounder in this study, an elaborately designed study with measurement at different gestational weeks would be better able to explain the association between inflammatory markers and full-term LBW. Sixth, despite the indirect effect explaining 48.4% of the total effect, the mediating effect of placental weight and maternal inflammatory markers on the associations between maternal SHS exposure during pregnancy and full-term LBW is only partial, rather than complete. Moreover, in contradiction with the previous finding that TNF-α may not cross the placenta^56^, our study found that TNF-α had a direct effect on full-term LBW without decreasing placental weight. Thus, other pathways, such as placental inflammation and vascular pathology, may lead to more substantial mediation of the effect of maternal SHS exposure during pregnancy on full-term LBW. Seventh, we estimated gestational age using the last menstrual period; however, as this measure may be inaccurate, more precise methods should be used to measure gestational age in future studies. Finally, we made the simplified assumption that the direction of the maternal-placenta-foetal effect was one way. As foetal growth can influence placental growth, this may also account for the partial mediation found in this study.

In summary, to the best of our knowledge, this is the first study to explore the pathways through which maternal exposure to SHS during pregnancy causes full-term LBW via maternal inflammatory mediators and damage to the placenta. Our findings support the above hypothesis and thus extend our understanding of the mechanisms of full-term LBW and provide necessary insights for the development of novel, effective and targeted immunomodulatory therapies to improve pregnancy outcomes.

## Methods

### Participants

The study population consisted of 390 pregnant women who gave birth to full-term (≥37 weeks) infants at the Maternity and Child Health Care Hospitals of Shenzhen and Foshan in Guangdong, China, from September 2009 to March 2010. Birth weight was measured on an electronic balance scale immediately after birth. Mothers whose infants weighed less than 2500 grams were categorised as full-term LBW cases. When a full-term LBW case was enrolled, a control was randomly selected from the mothers with newborns with a normal birth weight (2,500–4,000 grams) and gestational age of ≥37 weeks who went into labour on an adjacent date (+/−3 days) in the same hospital. Overall, 195 LBW cases and 195 controls were recruited in this case-control study. The study was approved by the Ethics Committee of Sun Yat-sen University in Guangzhou, China, and it was carried out in accordance with the approved guidelines. All of the participants understood the study and gave their written informed consent.

Mothers who had an infectious disease in the last trimester were excluded from the study to avoid bias in the measures of inflammation due to their infectious status. Women with the following characteristics or behaviour were not included: 1) actively smoked cigarettes during pregnancy; 2) had one or more pre-existing chronic renal diseases, lung disease, diabetes, hypertension, hyperthyroidism or anaemia; 3) had an induced or accidental abortion; 4) had multiple births or newborns with birth defects; 5) had one or more obstetric complications, such as pre-eclampsia, antepartum haemorrhage, placenta previa etc.; and 6) placenta calcification.

### Data collection

In the postnatal face-to-face interview, each selected mother was asked to complete a structured questionnaire on her social-demographic information, medical history, reproductive history, health behaviour and pre-pregnancy weight and height.

The medical records were reviewed to collect detailed gestational medical and obstetrical information including medication, last menstrual date, early ultrasound assessment, birth weight, infant gender, parity and placental weight.

### Measurement and definition of maternal SHS exposure

The information about maternal exposure to SHS during pregnancy was collected at the postpartum interview, as reported previously^57^. Briefly, a set of questions were asked: ‘Have you ever smoked even one cigarette?’, ‘Were you ever exposed to passive smoking during pregnancy?’, ‘If yes, how many minutes per day, on average, were you exposed to passive smoke during each of the first, second and last trimesters?’. The average maternal exposure to SHS during pregnancy was calculated as the arithmetic mean of the number of minutes per day in the three trimesters of pregnancy. Maternal exposure to SHS during pregnancy was defined as an average maternal exposure time of more than 0 minutes per day.

### Measurement of placental weight

The placenta, with membranes and large clots but with the cord trimmed, was weighed immediately after birth on an electronic balance scale in decagrams to the nearest 10 g. A lower placental weight was defined as below the median (500 g).

### Measurement of inflammation

Maternal blood samples were collected within 12 hours after the eligible mothers were admitted to the hospital for parturition, and they were not in active labour or membrane rupture. Two millilitres of maternal peripheral venous blood were collected in a serum separator tube and centrifuged at 3000 rounds/minute to obtain the serum. The serum specimens were immediately stored in a freezer at −80 °C until all of the samples were collected. Freezing and thawing cycles were avoided during the sample collection process.

The maternal serum levels of six inflammatory markers, MCP-1 (monocyte chemotactic protein-1), IL-6 (interleukin-6), TNF-α (tumor necrosis factor-α), IL-1β (interleukin-1β), CRP (C-reactive protein) and VCAM-1 (soluble vascular cell adhesion molecule-1), were measured using flow cytometric (bead-based) multiplex assays with the Human Basic Kit Flow Cytomix (a basic kit used in combination with BMS simplex kits to perform the quantitative detection of soluble human analytes by Flow Cytometry, Code: BMS8420FF, eBioscience, USA) and Flow Cytomix (CRP: BMS8288FF; VCAM-1: BMS8232/2FF; MCP-1: BMS8281FF; IL-1β: BMS8224FF; IL-6: BMS8213FF; TNF-α: BMS8223FF, eBioscience, USA) on a BD FACS Calibur instrument (BD Biosciences, USA), in strict accordance with the manufacturer’s instructions. Data were obtained using Cell Quest software (BD Bioscience) and calculated using the Flow Cytomix program (eBioscience, USA).

### Potential confounders

According to the literature^58^,^59^,^60^,^61^, we defined maternal age, education level, family income, pre-pregnant BMI, parity, newborn gender and gestational age as potential confounders in this study.

### Data analysis

Means and standard deviations (SD) were used to describe continuous variables. Levels of serum inflammatory markers were logarithmically transformed and described as geometric means with 95 per cent confidence intervals (95% CIs). Proportions were used to describe the distributions of categorical variables.

First, Chi-square tests were used to examine the associations between full-term LBW and maternal social-demographic and clinical characteristics, and Student’s *t* tests or Satterthwaite *t*-tests were performed to test the differences in maternal age, gestational age, placental weight and log-transformed maternal serum levels of inflammatory markers between the full-term LBW group and the normal control group.

Second, logistic regression analysis was performed to test the associations between full-term LBW and maternal exposure to SHS during pregnancy, lower placental weight and maternal serum levels of inflammatory markers, with adjustment for potential confounders. Partial correlation analyses were conducted to examine the correlations between any two of the maternal inflammatory markers while controlling for the potential confounders.

Third, SEM was conducted to explore the potential pathways of maternal inflammation and lower placental weight in the association between maternal exposure to SHS during pregnancy and full-term LBW, on the basis of the fit between our data and the hypothesised pathways^54^,^55^. The model fit was evaluated by the root mean square error of approximation (RMSEA) ≤ 0.10, goodness of fit index (GFI), incremental fit index (IFI) and comparative fit index (CFI) ≥ 0.90^62^,^63^.

All *P* values were two-sided and statistical significance was set at *P* = 0.05. The structural equation model was calculated using AMOS version 17 (SPSS Inc. Chicago, Illinois, USA), and all of the other statistical analyses were conducted with SPSS version 17.0 (SPSS Inc. Chicago, Illinois, USA).

## Additional Information

**How to cite this article**: Niu, Z. *et al.* Potential pathways by which maternal second-hand smoke exposure during pregnancy causes full-term low birth weight. *Sci. Rep.* **6**, 24987; doi: 10.1038/srep24987 (2016).

## Figures and Tables

**Table 1:** Comparison of socio-demographic and obstetric characteristics in full-term LBW and control.

	Full-term LBW (N = 195)	Control (N = 195)	t/χ^2^	*P*
Maternal Age, Mean ± S.D. (years)	28.79 ± 4.53	29.40 ± 4.21	1.39	0.170
Gestational Age, Mean ± S.D. (week)	38.03 ± 1.01	39.08 ± 1.18	9.41	<0.001
Birth Weight, Mean ± S.D. (g)	2343.87 ± 184.44	3300.99 ± 374.02	32.12	<0.001
Placenta Weight, Mean ± S.D. (g)	466.30 ± 55.79	502.01 ± 39.29	7.31	<0.001
Race/Ethnicity, n (%)			0.07	0.780
Han	187 (95.9%)	188 (96.4%)		
Minority	8 (4.1%)	7 (3.6%)		
Marital Status, n (%)			0.64	0.424
Married	186 (95.4%)	189 (96.9%)		
Unmarried	9 (4.6%)	6 (3.1%)		
Education Level, n (%)			5.93	0.052
Junior high school or lower	60 (30.8%)	40 (20.5%)		
High school	49 (25.1%)	50 (25.6%)		
College or higher	86 (44.1%)	105 (53.9%)		
Family average income per month, n (%)			3.22	0.073
Less than 490$ per month	87 (44.6%)	70 (35.9%)		
Over $490 per month	108 (55.4%)	125 (64.1%)		
Pre-pregnancy BMI, n (%)			1.87	0.392
Normal (18.5~23.9kg/m^2^)	123 (63.1%)	128 (65.7%)		
Underweight (~18.4kg/m^2^)	59 (30.2%)	49 (25.1%)		
Overweight or obesity (≥24kg/m^2^)	13 (6.7%)	18 (9.2%)		
Parity, n (%)			1.10	0.294
Nulliparous	154 (79%)	145 (74.3%)		
Parous	41 (21%)	50 (25.7%)		
Infant Gender, n (%)			5.49	0.019
Male	79 (40.2%)	101 (51.8%)		
Female	116 (59.8%)	94 (48.2%)		

**Table 2:** Association between maternal SHS exposure, placental weight, maternal serum levels of inflammatory markers and the full-term LBW^#^.

	Maternal SHS exposure (OR; 95% CI)	Lower Placental Weight (OR; 95% CI)	Partial correlation coefficient (*pr*)						The full-term LBW (OR; 95% CI)
			MCP-1	IL-6	TNF-α	IL-1β	CRP	VCAM-1	
Maternal SHS exposure (No vs. Yes )	1								2.14^*^ (1.06~4.32)
Placental Weight **(≧**500g vs. <500g)	2.30* (1.10~4.81)	1							3.60^*^ (2.09~6.19)
MCP-1	1.07 (0.66~1.74)	0.83 (0.60~1.16)	1						0.82 (0.59~1.14)
IL-6	1.07 (0.82~1.40)	1.15 (0.96~1.38)	0.19^*^	1					0.99 (0.83~1.17)
TNF-α	1.55^*^ (1.06~2.27)	1.28^*^ (1.01~1.63)	0.03	−0.14^*^	1				1.92^*^ (1.47~2.50)
IL-1β	1.72^*^ (1.11~2.66)	1.35^*^ (1.00~1.80)	0.05	0.01	0.25^*^	1			1.53^*^ (1.14~2.05)
CRP	1.21 (0.92~1.59)	1.00 (0.86~1.17)	0.03	0.11^*^	0.12^*^	0.11^*^	1		0.99 (0.85~1.15)
VCAM-1	1.28 (0.91~1.81)	0.76^*^ (0.62~0.95)	0.07	−0.14^*^	0.14^*^	−0.05	0.12^*^	1	0.85 (0.69~1.04)

^#^Adjusting for maternal age, educational level, family income, pre-pregnant BMI, parity, and pregnant weeks.

^*^P < 0.05.

**Table 3:** Effect of maternal SHS exposure during pregnancy on full-term LBW.

	Path		Effect	Proportion to total effect
Direct	1	SHS→ LBW	0.066	51.6%
Indirect	2	SHS→ IL-1β→…VCAM-1 → placenta →LBW	0.031	24.2%
	3	SHS→ IL-1β→ TNF-α→ LBW	0.031	24.2%
Total			0.128	100.0%
